# An operations research approach to automated patient scheduling for eye care using a multi-criteria decision support tool

**DOI:** 10.1038/s41598-022-26755-1

**Published:** 2023-01-11

**Authors:** Luke Evans, Jennifer H. Acton, Carla Hiscott, Daniel Gartner

**Affiliations:** 1 School of Mathematics, Cardiff University, Cardiff, UK; 2 School of Optometry and Vision Sciences, Cardiff University, Cardiff, UK; 3 Aneurin Bevan University Health Board, Newport, UK

**Keywords:** Mathematics and computing, Applied mathematics, Health care, Health policy, Health services

## Abstract

Inefficient management of resources and waiting lists for high-risk ophthalmology patients can contribute to sight loss. The aim was to develop a decision support tool which determines an optimal patient schedule for ophthalmology patients. Our approach considers available booking slots as well as patient-specific factors. Using standard software (Microsoft Excel and OpenSolver), an operations research approach was used to formulate a mathematical model. Given a set of patients and clinic capacities, the model objective was to schedule patients efficiently depending on eyecare measure risk factors, referral-to-treatment times and targets, patient locations and slot availabilities over a pre-defined planning horizon. Our decision support tool can feedback whether or not a patient is scheduled. If a patient is scheduled, the tool determines the optimal date and location to book the patients’ appointments, with a score provided to show the associated value of the decisions made. Our dataset from 519 patients showed optimal prioritization based on location, risk of serious vision loss/damage and the referral-to-treatment time. Given the constraints of available slots, managers can input hospital-specific parameters such as demand and capacity into our model. The model can be applied and implemented immediately, without the need for additional software, to generate an optimized patient schedule.

## Introduction

Healthcare systems are under increasing pressure due to the rising demand and expenditures for healthcare. This problem is compounded by the increase in the elderly population together with the increased global burden of vision impairment^1^. In Wales, UK, the total number of patients in the highest risk category waiting for an outpatient appointment was over 130,000, in January 2022^2^. It is predicted that a 40% rise in the number of ophthalmologists will be required to meet eye care demands by 2040^3,4^. This is due in part to the ageing population and in part to the greater availability of treatments. The COVID-19 pandemic has intensified the need to address this problem^5^.

As a result of the COVID-19 pandemic, there have been increased breaches in waiting time targets for ophthalmology outpatient clinics^6^. Prior to the pandemic, such waiting times were already undesirably high due to the backlog resulting from the vast number of patients requiring treatment, compounded by existing inefficient scheduling practices^7,8^. This is further complicated by the ever-present budgetary restrictions on health services. Missed or delayed appointments of high-risk patients could be mitigated by adopting a transparent and efficient scheduling process for all patients who need treatment. The management of such hospital appointments is typically implemented using a manual, inefficient and time-consuming approach to book patients into available slots.

Manual patient scheduling aims to minimize the time between referral and treatment (referral-to-treatment time; RTT^9^) for patients booked into available appointment slots, thus prioritizing those with longer, over those with shorter, waiting times. Such a system does not account for the risk of vision loss associated with delay^10^ and is subject to the potential human errors and inconsistencies associated with a manual process which can cause inefficient time slot allocation. When examined on a large scale, many patients that require immediate care may not be seen until the queue in front of them has subsided, increasing their risks associated with further delay.

Mathematical formulation has been used to provide a solution to the planning problem with input to daily scheduling, with the use of capacity-based constraints^11^ and incorporation of inherent uncertainty from late cancellations^12^. A ‘rolling horizon’ or a pre-defined time window in which to book appointments^13^ has also been used. Previous studies have employed computer modelling using discrete event simulation and optimization methods to improve patient scheduling^14–16^ as well as time-motion analysis to manage workflows^17^. Others have applied stochastic programming to patient scheduling, an optimization method which accounts for uncertain parameters^18–21^. Some studies have employed simulations^18,21,22^. One such approach showed simulations involving appointment data to allocate slots in clinics whereby the estimated length of the appointment is the main factor in determining when a patient is to be seen^22^. Others have proposed decision support systems that provide real-time and dynamic assistance to human schedulers based on probability^23^.

In contrast to previous work, the aim of our study is to optimize the scheduling of ophthalmology patients using an operational research approach that will take the available slots for treating individuals and allocate them based on their location, risk of serious vision loss/damage and the referral-to-treatment time. A tool was developed with the ability to prioritize patients based on these factors and the needs of the user to create a schedule for the patients, using the availability of treatment slots, that can be utilized in the absence of extra, clinic specific, constraints. The tool is designed for use on standard software, maximizing the usability and acceptability in practice. Another novelty of our approach is that a decision maker can find a trade off between these multiple objectives.

A problem statement is described in the methods section (see “Problem statement and algebraic model formulation”).

## Results

Table 1 provides an overview of the parameters used in the study, derived from the real-world clinical data, for implementation of the scheduling tool.

**Table 1:** Parameters for the analysis and data from hospital clinics.

Number of patients	519
Total slot capacity	329
Number of clinics	3
Time horizon (weeks)	6

### Summary of models

Table 2 shows the outcome of the model using the data from Table 1, from 519 patients with their corresponding RTT and risk scores. The number of patients not scheduled computes as $P-\left({\sum }_{p=1}^{P}{\sum }_{l=1}^{L}{\sum }_{t=1}^{T}\left({x}_{p,l,t}\right)\right)$. This means that we subtract the number of scheduled patients from the total number of patients $P$. In order to get the remaining RTT or risk score for patients that we introduce a 1-dimensional binary auxiliary variable that indicates whether a patient has or has not been scheduled. We then multiply this vector by the patient-individual $RT{T}_{p}$ or ${R}_{p}$ value, respectively and calculate the sum product between the auxiliary variables and the parameter vector.

**Table 2:** Model outcome showing the results by maximizing the individual and combined objectives (column objective).

Objective	Performance metrics						
	Total number of weeks from referral to treatment (RTT objective)	Total distance score (distance objective)	Total risk (risk-objective)	Number of patients scheduled	Sum of remaining RTT for patients that were not scheduled	Sum of remaining risk score for patients that were not scheduled	Number of patients not scheduled
RTT	8746	8131	134,798	329	2926	102,790	190
D	7548	14,554	150,665	329	4124	86,923	190
R	7344	8751	235,940	329	4328	1648	190
RTT + D	8168	14,250	143,708	329	3504	93,880	190
D + R	7363	13,404	235,940	329	4309	1648	190
RTT + R	7843	8595	235,940	329	3829	1648	190
RTT + D + R	7658	13,306	235,922	329	4014	1666	190

The first line reveals a solution in which the model is optimized using RTT with the maximum of 8746 weeks (which equates to an average of 26.58 weeks per patient) spread across 329 scheduled patients. Note that optimizing using the risk-oriented objective in combination with distance, or RTT has the biggest impact on scheduling high-risk patients. The distance score is in that solution, however, as low as 8751 which can be boosted to 13,404 by combining the risk and distance objectives and keeping the risk reduction at the same value. None of the high risk patients remained in the 190 individuals who were not scheduled. The scheduling model provides information about both weeks and days: the total RTT reduction from the waiting list (in weeks) plus the day when a patient has their appointment as determined by the variables input into the model.

The derivation of the objective functions is based on the selected factor or combination of factors from RTT, distance or risk of scheduled patients, added together to produce a summed score that is referred to as the objective. It is desirable to maximize each of the three factors associated with the patients scheduled, in order to prioritize patients with higher scores. Therefore, the higher the objective scores, the better the solution in terms of the optimization objective.

Table 2 shows the scores generated for RTT, distance and risk when the model is given the objective function, or combination objective functions, shown on the leftmost column to maximize. Using an objective function that incorporates multiple separate parameters summed together will be equivalent to deciding that these selected parameters have equal weighting that is higher than those not selected. For example, in prioritizing risk alone (labelled in Table 2 as ‘R’), the model will maximize the risk objective and produce a score of 235,940, whilst the objective functions of RTT and distance, labelled here as D, produce scores of 7334 and 8751 respectively. On the other hand, if we were to choose the objective function that assigns equal weighting to distance and risk, i.e., prioritizes distance and risk, then the three base objective functions will produce scores of 7363, 13,404 and 235,940.

**Figure 1:**
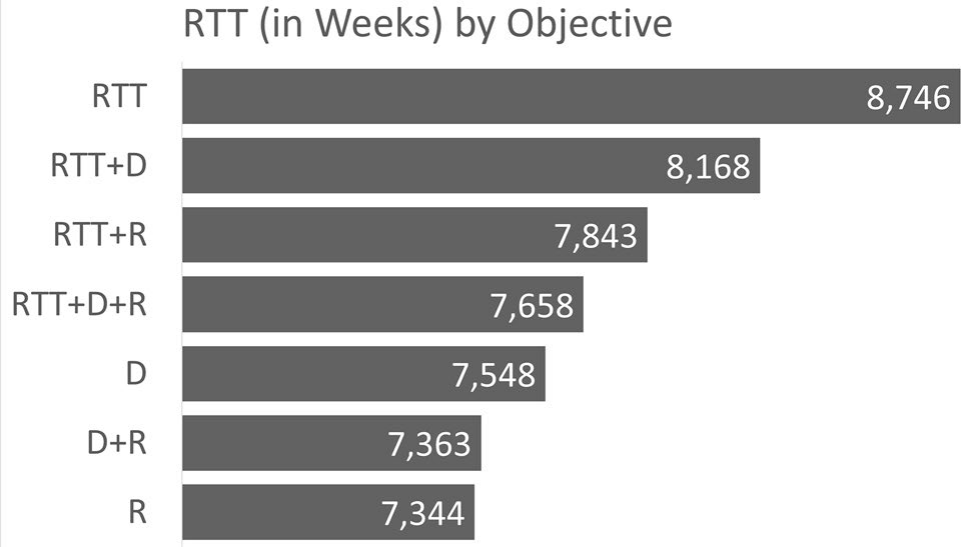
Bar chart representing the RTT measure broken down by models.

**Figure 2:**
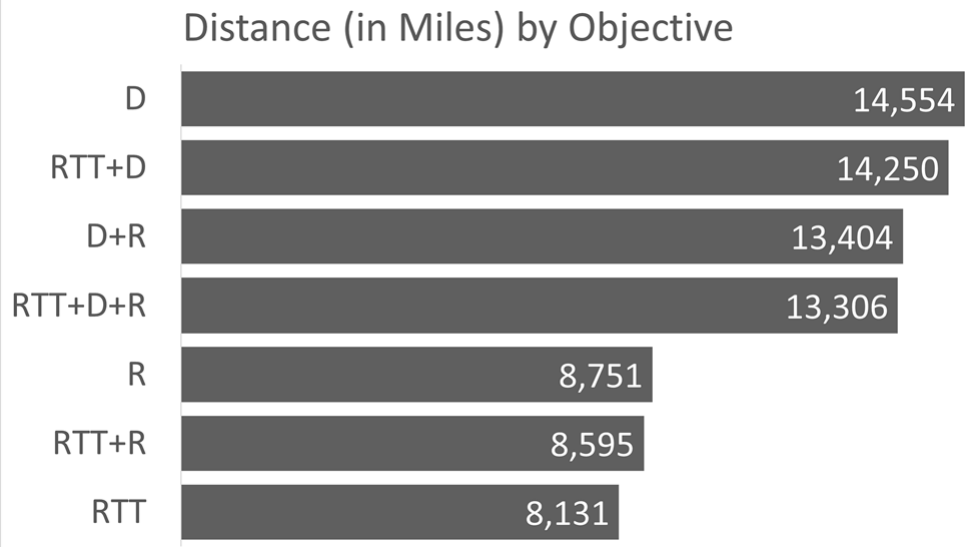
Bar chart representing the distance measure broken down by models.

**Figure 3:**
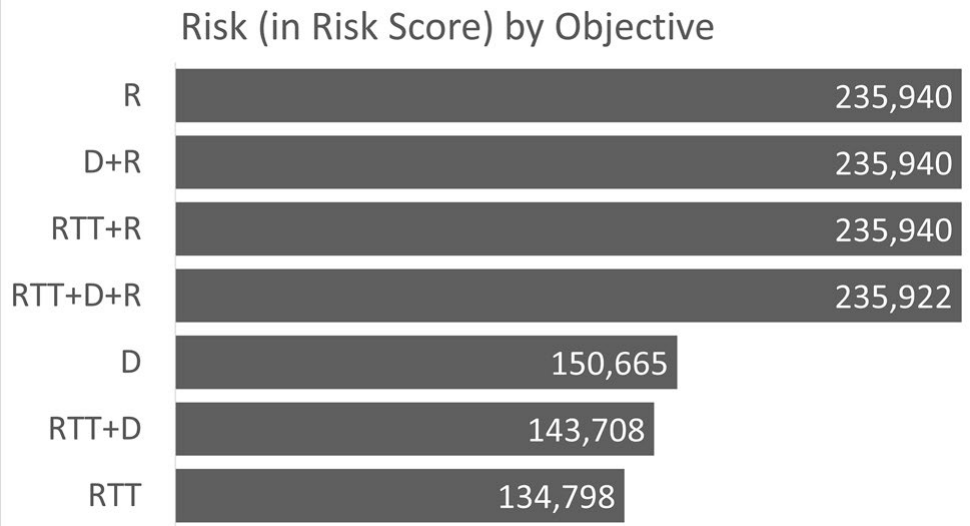
Bar chart representing the risk measure broken down by models.

In Fig. 1, an overview of the RTT reduction is provided and broken down by each model that we solve. For example, solving the model with the objective to minimise RTT, the RTT reduction (in weeks) is 8746. In contrast, if the risk-related objective is pursued, then we have an RTT reduction of only 7344 weeks. Similarly, Figs. 2 and 3 provide an overview of the distance reduction and risk reduction, respectively. Evidently, if risk reduction is the dominating objective, and in addition, distance or RTT reduction was the secondary objective, then the decision maker should use the D + R or RTT + D model, respectively.

Note that the overall lowest risk score occurred when RTT was prioritized. Therefore, if risk factor is the objective that the user wishes to optimize, then it must be allocated a weighting that is higher than at least one other contributing factor in the model. The outcome of prioritizing all factors with equal weightings (i.e. RTT + D + R) resulted in an extremely close, but slightly lower score (235,922) as can be seen in Fig. 3.

In each case, the maximum capacity of patients was reached, as shown in Table 1 (and in Fig. S1 Supplementary Information), which can be seen by comparing the number of patients allocated by the model to the capacities in each location. The outcome implies that the solutions from each objective function are optimal as the model does not leave any spaces to be filled. By ensuring that every slot is filled we can ensure that the model cannot miss patients, which is a result of maximizing the values of each objective.

## Discussion

The tool developed in this study can be used to schedule patient appointments in ophthalmology clinics, using standard software that is already available. The tool is based on real-world data and is able to create feasible schedules as long as the capacities of the clinics for each day can be reliably met. Testing of the model shows that an optimal patient appointment schedule can be met whilst accounting for the key factors of distance between patients and clinics, RTT dates and individual patients risk factors, the former is becoming increasingly important due to government guidelines concerning the delivery of care closer to home^24^. Utilizing this tool will help reduce the effect that these factors have on the care of patients in order of priority that is to be determined by the user, or simply all factors together at once.

In order to apply the model in practice, schedulers would use the tool similar to a rolling-horizon planning fashion^25^. Every day, patient requests would be collected and added to the patient waiting list. Then, at the end of the day, patients scheduled from the list would get a notification about their day and location of appointment. This can also be done on a weekly basis i.e., collecting patient requests until the end of a week and then at the beginning of the new week, patient notifications would be sent to the patients.

In recent years factors such as ageing populations and the improved early detection of medical issues have highlighted the importance that hospital capacities are met, and new ways of solving the problem are required. With the presence of the COVID-19 pandemic, this problem is only becoming more evident amongst healthcare providers^26–28^, due to the rapid influx of patients^29^. Therefore, the increased use of tools, such as the one described in this study, that incorporate an operations research approach would help to streamline patient scheduling in medical departments. The tool presented here is flexible in that it can be adjusted to apply to different scenarios. It has a fast running time, within seconds, and is scalable for larger datasets.

The implementation of operations research in healthcare has received much attention in recent years^30–32^. Operations research models have been implemented previously in patient scheduling^33^. A common theme in such studies is that the goal is to optimize the resources of a clinic in each day^34^ reduces patient wait times. The tool described in the present study differs to the previous work in that it determines which patients should be seen first and can account for a backlog of patients that cannot be seen on a particular day. In this study, changing the objective that the user wishes to optimize can greatly change the final result of the model. Based on data from three hospital clinics in Wales, our results show that the optimal method of generating the highest overall score from the objective functions is to give all three factors equal weighting. In the computational study, risks R1, R2 and R3 were given values of 1000, 10 and 1, respectively. As a consequence, this objective is prioritized if the RTT or distance does not exceed 1000 (weeks or miles). Scaling these values differently may lead to different solutions. This reflects the feature that the patients who qualify for the highest risk category are those who require treatment as soon as possible, therefore the goal of prioritizing high-risk patients is upheld. Automated systems implemented in patient scheduling are advantageous relative to a manual approach^11,12,16,20,35–38^.

The study does not evidence the performance or need of the proposed tool. There are further limitations to this study: (1) the scheduling tool does not allow for allocating patients a specific time slot on a given date; (2) the model assumes that the number of patients that can be seen stays at the capacity and so does not account for changes in staff, for example due to sick leave; (3) if a patient misses the appointment given, re-allocation would require running the model again.

The above limitations can be overcome. Many studies have been conducted on the topic of scheduling patients to specific time slots^39,40^ and if the user so desired, it would be possible to create an additional model, similar to the one presented here, that is to be used after the original model has been run. This new model would use specific time slots in a day for a given clinic instead of separate days and the data would have to change from clinic capacities to the number of patients that can be seen to at the corresponding time slot.

Issues related to capacity variations would be mitigatable in the real-world application of the model by the planning of the user. This ensures that changes in the number of staff and treatment availability can be assumed to be negligible when creating a scheduling tool as accounting for such variations can add a layer of complexity to the model. Therefore, such complexity is a potential barrier to the accessibility of the tool.

To reduce the number of patients that miss their scheduled appointment date, allowing patients to select a specific date is possible within the model in this study. Alternatively, a user would be able to run the model and inform patients scheduled to the first available date before any others, in order to determine whether or not the date is acceptable, run the model again with the confirmed patients already allocated and repeat the process until the schedule is complete.

Another open question is how to scale the risk score and how to provide a normalised comparison of the risk score against RTT and distance, if the objective functions are combined. In this study, we have chosen 1, 10 and 1000 for low, medium and high risk score, respectively. In our particular case this avoids that scheduling for example 200 patients with medium risk score outweigh one patient scheduled with high risk score. Flexibility is, however, provided for low and medium risk score where we would allow multiple patients scheduled with a low risk score may outweigh patients scheduled with a medium risk score. This is, however, up to the discretion of the scheduler whether it is, from a clinical point of view, acceptable that this outweighing behaviour takes place. Another issue is the comparison between the different measures. When we combined the objectives in the case study, we gave each of the objectives the same weight. However, a healthcare organisation may have different priorities how to weight each of the objectives. For example, it might be better to give the risk reduction the highest weight, give RTT reduction the second highest weight and the distance minimization a low weight.

In summary, the tool created for this study will allow users to streamline their patient scheduling in order to allow for priority to be given under specified circumstances, without the need to obtain specialist software. This study used data gathered from hospital clinics in the UK to provide an example of how the tool can be implemented in a set of clinics, over a specified time horizon, to show how multiple different objectives, or weighted combinations of them, can be maximized in order to optimize the specific needs of the user. Future work involving comparing the optimization presented here with current manual scheduling will help to inform the utility, validity and sustainability of the tool, as well as the applicability to other areas of healthcare. Consideration of the cost-effectiveness of the tool would address the need for easily scalable strategies given increasing demands on services and limited resources, as well as evaluation of the potential to improve health outcomes. As a consequence, the tool has the potential to help clinics to improve their service and ensure that key patients are seen before their condition gets worse, improving the overall quality of care that can be achieved.

## Methods

We approach the problem of planning the patient schedule of ophthalmology clinics using an Integer Linear Programming formulation^41^, in which a real-world problem is expressed in terms of linear inequalities. The model was designed using the Microsoft Excel add-in OpenSolver. Anonymized data were obtained from three National Health Service hospital eye clinics in Wales and included the capacities and patient numbers from the clinics. The protocol was approved by the Aneurin Bevan University Health Board (ref no. SA/1272/21), who granted permission for access to data and all methods were carried out in accordance with the relevant guidelines and regulations. Informed consent was not required on the basis that all data were fully anonymised.

The model includes, over a predetermined planning horizon: a given number of patients that each have an assigned referral-to-treatment time, a given distance factor between the patient and the clinics, and associated risk factor, which is to be determined by the patient’s treatment provider. The model is designed to allocate patients to time slots within clinics that have fixed patient capacities for each day. The model assumes shared electronic medical records between clinics.

In addition to the real data used by the model, further data to represent the distance travelled by each patient were sampled from a uniform distribution with lower and upper bounds of 1 and 50, respectively. These numbers represent a ‘distance score’ rather than a precise distance as it is desirable to design the model to maximize each objective function, rather than minimizing exact distances. Therefore, a higher ‘distance score’ indicates the patient is located closer to the specific clinic. In application, these scores will be replaced by the appropriate distance scores of each patient individually. The correlation between distance score and real numerical distance can be determined by the decision maker based on how far the range of patient travel distances is, i.e., in some cases patients may all be within a mile of all clinics whereas in other cases they may be within 10 miles of clinics.

The risk factor for a patient is defined in different categories by eye care services in Wales^42^. R1 indicates “risk of irreversible harm or significant patient adverse outcome if target date is missed”, e.g., neovascular age-related macular degeneration; R2 indicates “risk of reversible harm or adverse outcome if target date is missed”, e.g., cataract; and R3 as “no risk of significant harm or adverse outcome”, e.g., eyelid lesion with no malignancy suspected. In the model R1, R2 and R3 were represented by 1, 100 and 1000, respectively, as these values, when used in the model, give adequate numerical separation, to appropriately prioritize those in higher risk categories.

The model assumes that patients can be treated within a standard appointment duration^43^, allowing for the use of clinic capacities instead of minute-by-minute time scheduling. The model considers:

- Patient referral-to-treatment (RTT) time
- Risk factor associated with patient’s treatment being further delayed (R1, R2, or R3)
- Clinic capacities
- Distance score that corresponds to the distance that the patient must travel to reach the clinic
- Relative weighting of the above objectives for prioritization, depending on the specific circumstances and user’s needs

### Decisions to be made by the model

The model is able to decide the clinic and date that the patient is to be allocated within the time horizon. Consequently, the model will decide who will be seen and, if capacity is scarce, who must still wait. As the clinics have fixed capacities for each day it will be the decision of the clinic staff to provide the specific time at which the patient is to be seen. Depending on the requirements of the user, the planning horizon can be made longer or shorter. In this study, the model is applied using patient numbers that exceed clinic capacities to ensure that the prioritization of patients at greatest risk.

### Objective functions and constraints

The model allows the user to maximize the score associated with a given patient’s risk factor, in order to prioritize those who have the largest risk of serious damage as compared to patients with a low eye care risk. This was undertaken using a large binary matrix that indicates for each patient whether or not they will be allocated a unique clinic. The binary values are multiplied by, depending on the objective, the RTT, distance or eye care risk score and then summed to give the final objective value. The constraints of the model ensure that patients cannot be allocated to more than one slot and that the total number of patients scheduled cannot exceed the clinic’s capacity for a given date. Although the model considers a unique objective (RTT, distance or eye care risk score), it can also allow for the user to find a trade off between the measures by using a combination of two, or all three objectives. This, in practice, meets the different demands of specific users.

### Problem statement and algebraic model formulation

In order to state the problem, we introduce the sets, indices, and other parameters to algebraically state the problem. Let ${\mathcal{P}}$ denote the set of patients and let ${\mathcal{T}}$ denote the set of days with $T$ representing the last day in the planning horizon in which patients can be assigned to clinics. For example, ${\mathcal{T}}: = \left\{ {1,2,3 \ldots ,T} \right\}$ represents the set of days labeled as day 1, day 2 etc. until the last day $T$. Next, we have a set of locations ${\mathcal{L}}$ where clinics take place. We also introduce ${C}_{l,t}$ as the capacity of a clinic at location $l\in {\mathcal{L}}$ at day $t\in {\mathcal{T}}$. Patient-dependent parameters are $RT{T}_{p}$ which denote the referral-to-treatment time of patient $p\in {\mathcal{P}}$. Furthermore, ${R}_{p}$ denotes the eye-care risk measure of patient $p\in {\mathcal{P}}$. Finally, ${D}_{p,l}$ denotes the distance (score) that patient $p\in {\mathcal{P}}$ has to travel if their appointment is scheduled at location $l\in {\mathcal{L}}$.

We introduce binary decision variables ${x}_{p,l,t}$=1 if patient $p\in {\mathcal{P}}$ has a scheduled appointment at location $l\in {\mathcal{L}}$ at day $t\in {\mathcal{T}}$ and 0 otherwise.

We consider three objective functions to schedule patients based on their RTT value, their risk measure, and distance to the clinics denoted as RTT, Risk, and Distance, respectively:

$${\text{Maximize}}\sum_{p\in {\mathcal{P}}}\sum _{l\in {\mathcal{L}}}\sum_{t\in {\mathcal{T}}}RT{T}_{p}\cdot {x}_{p,l,t}\qquad (\text{RTT})$$

$${\text{Maximize}}\sum_{p\in {\mathcal{P}}}\sum _{l\in {\mathcal{L}}}\sum_{t\in {\mathcal{T}}}{R}_{p}\cdot {x}_{p,l,t}\qquad (\text{Risk})$$

$${\text{Maximize}}\sum_{p\in {\mathcal{P}}}\sum_{l\in {\mathcal{L}}}{\sum }_{t\in {\mathcal{T}}}{D}_{p,l}\cdot {x}_{p,l,t} \qquad(\text{Distance})$$

The constraints are:

1 $$\sum_{t\in {\mathcal{T}}}{x}_{p,l,t}\le 1 \quad \quad \forall p\in {\mathcal{P}}$$

2 $$\sum_{p\in {\mathcal{P}}}{x}_{p,l,t}\le {C}_{l,t} \quad \quad \forall l\in {\mathcal{L}},t\in {\mathcal{T}}$$

3 $${x}_{p,l,t}\in \left\{{0,1}\right\} \quad \quad \forall p\in {\mathcal{P}},l\in {\mathcal{L}},t\in {\mathcal{T}}$$

Constraints (1) ensure that a patient is not scheduled more than once at a location while Constraints (2) ensure that the location- and day-dependent capacity is not exceeded. Expression (3) are the decision variables and the domains.

**Figure 4:**
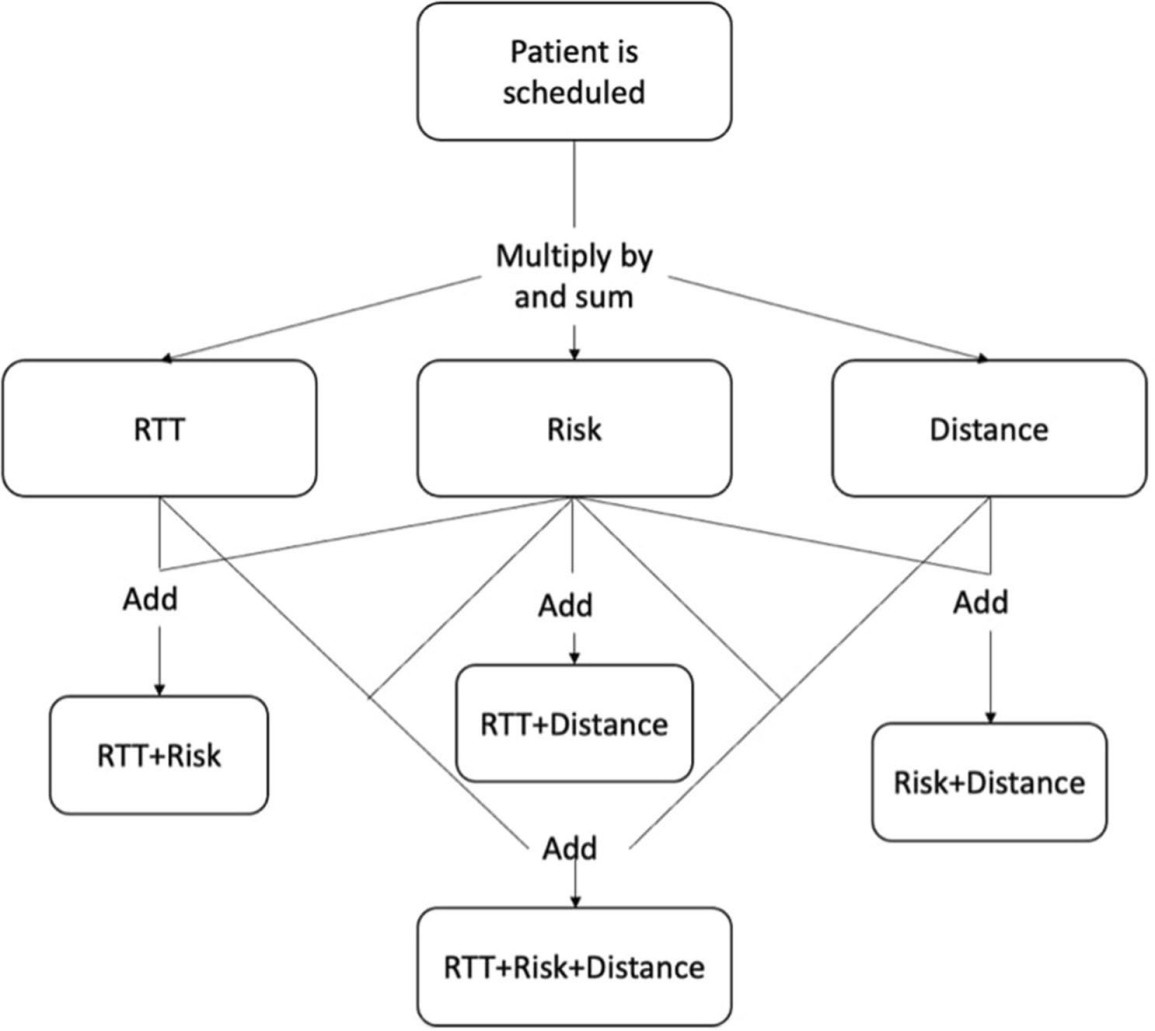
Schematic of model showing how the objective functions are formed.

Fig. S2 (Supplementary Information) shows how the objective function and constraints are entered into the solver. There is the possibility to use either one objective or to aggregate multiple objectives. The objective cell will be the value that we want to maximize, such as reduction in the total of patient risk factor values. In this iteration of the model the user has the option of seven objectives, as shown in Fig. 4.

As can be seen, the model uses binary values to represent the decision variables. These are then aggregated in the individual objective function. These objectives are the sum of different values, i.e., the relevant values are added together. The RTT, Distance, and Risk can also be explained as follows:

- RTT—the RTT number associated with each patient that has been scheduled summed.
- Distance—the sum of the distances the patients travel to the slot/clinic that they have been assigned.
- Risk—the total risk score that a clinic accumulates in each day by scheduling patients is calculated.

The model is able to provide a solution which allows for prioritization of each of the above objectives, in addition to a combination of any two, or all three, of these, giving seven possible prioritization outcomes/ combinations to choose from. The user can decide which of these seven objectives they wish to use by changing the objective cell in the model.

The variable cells will be the cells that correspond to where the patients will be allocated. The data should be structured in Microsoft Excel such that, a row is allocated to each patient and a column to each possible allocation location (see Fig. S3 in Supplementary Information). Constraints will determine as to the values that the model can generate for these cells and these cells in turn will give the objective functions when combined with the relevant data in the objective function formulas.

In the example ‘Solver’ outcome in Fig. S3, each row represents a different patient and each column is a possible allocation for the patient, i.e. clinics A, B and C on dates 1,…,6. The objective function then aggregates all decisions i.e. whether patients are scheduled or not and multiplies them with the corresponding RTT, risk or distance measure.

The first constraint ensures that the values in the variable cells will be ‘1’, i.e., the patient is allocated this slot, or ‘0’, i.e., no allocation. The second constraint ensures that a patient cannot be given more than one appointment and the final constraint ensures that the number of patients scheduled to a clinic on a specific date cannot exceed the given capacity. Here, the number of patients scheduled is the sum of values in each column. As a result of the first two constraints, each patient can have at most a single ‘1’ in each row.

## Supplementary Information

Supplementary Information.

## Data Availability

The dataset generated during the current study is available from the corresponding author on reasonable request.
