# Supplementary material for: An operations research approach to automated patient scheduling for eye care using a multi-criteria decision support tool
Source: Sci Rep. 2023 Jan 11;13:553. doi: 10.1038/s41598-022-26755-1 (PMC9832406; doi:10.1038/s41598-022-26755-1)
Supplement: Supplementary file 1 — Supplementary Information. [file 41598_2022_26755_MOESM1_ESM.docx]

**Supplementary information**


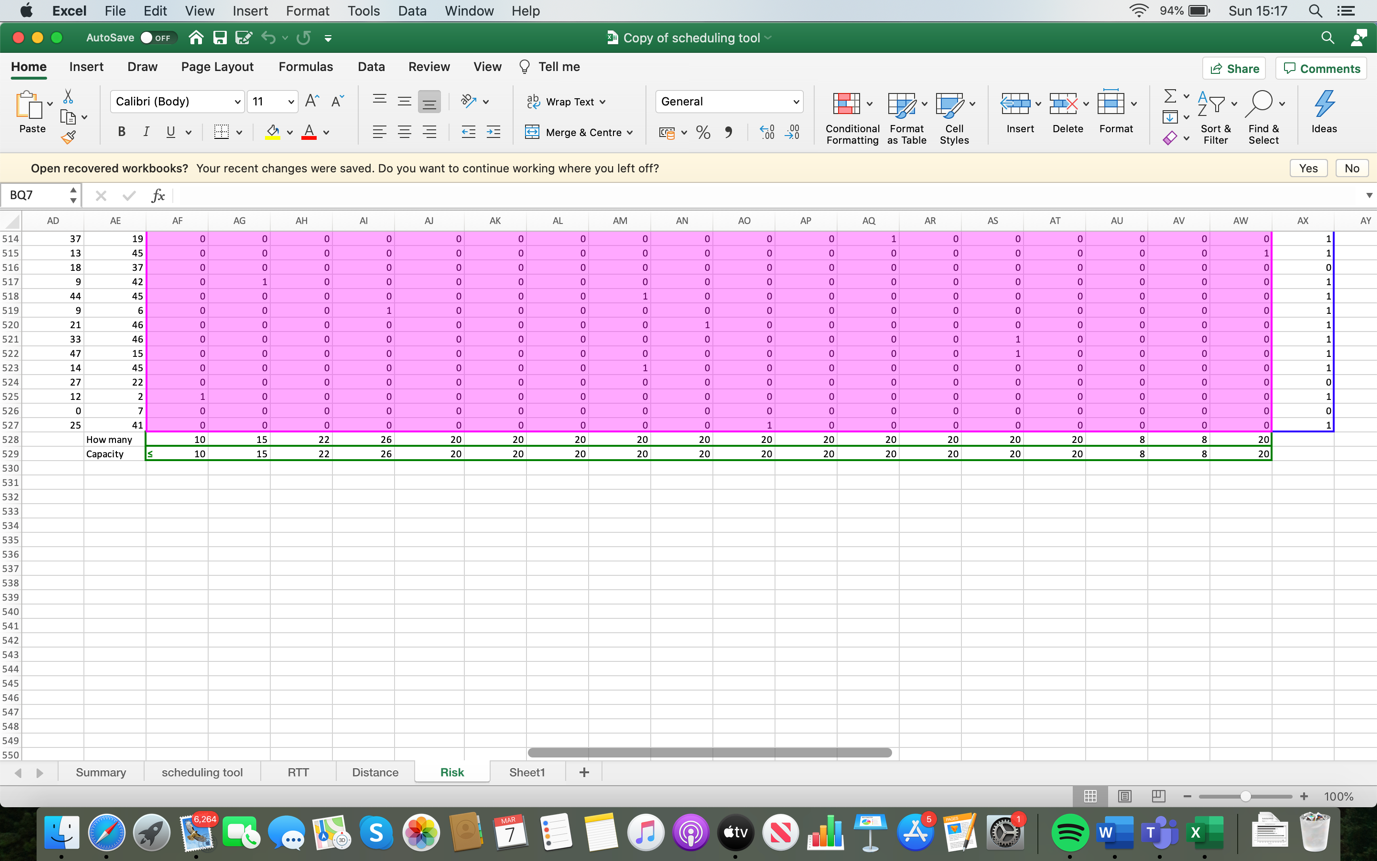


Figure S1: Example solution with constraints in practice


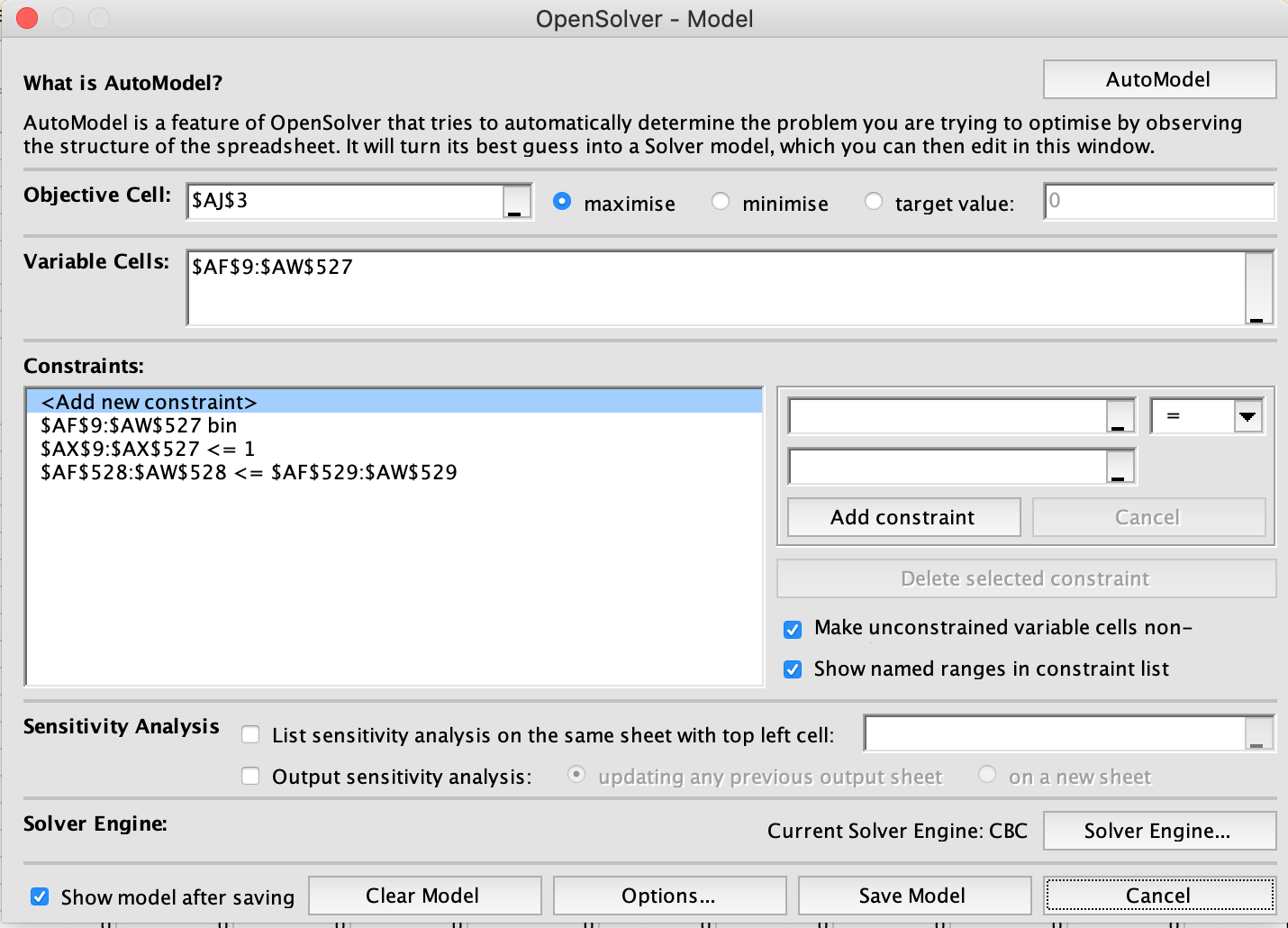


Figure S2: OpenSolver model. A screenshot from ‘Solver’ demonstrating the application of the model.


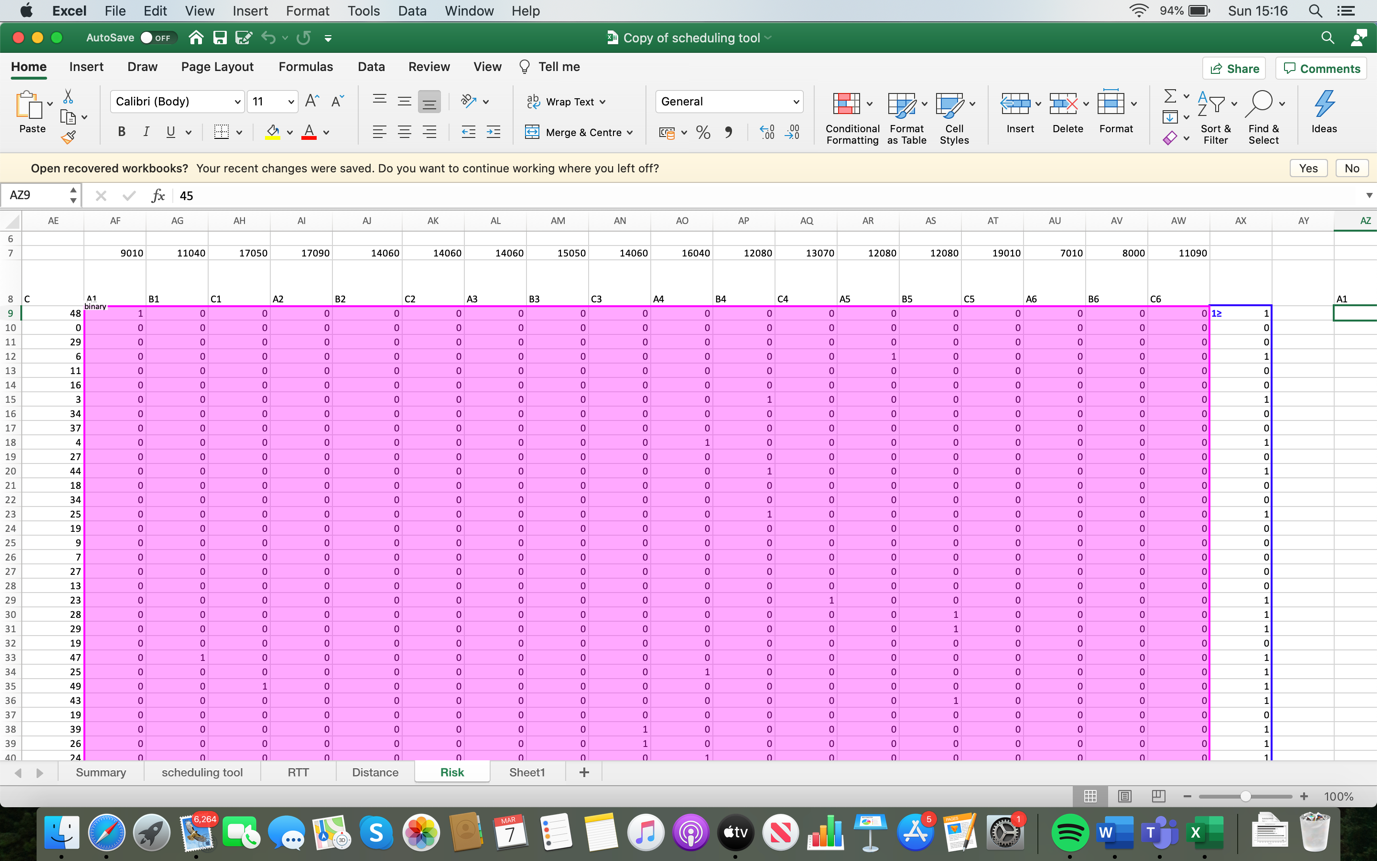


Figure S3: Example of results in an Excel sheet. Each row represents a patient and each column represents a slot for patients to be allocated, in which ‘1’ indicates a slot is allocated and ‘0’ indicates no allocation. The final column shows the sum of the values generated each row.
